# Lipoprotein(a) and Triglycerides as Independent Predictors of Long-Term Aortoiliac Stent Failure After Endovascular Revascularisation

**DOI:** 10.3390/jcdd13100513

**Published:** 2026-10-11

**Authors:** Bibombe Patrice Mwipatayi, Bernardette Jingfei Lee, Davina Dauda, James Evan Dodd, Mariah Theresa Mwipatayi, Jonathan Golledge, Trevor Anthony Mori, Gerald Francis Watts, Markus Peter Schlaich

**Affiliations:** 1 School of Surgery, University of Western Australia, Perth, WA 6000, Australia; 2 Department of Vascular and Endovascular Surgery, Sir Charles Gairdner Hospital, Perth, WA 6009, Australia; 3 Department of Vascular and Endovascular Surgery, Royal Perth Hospital, Perth, WA 6009, Australia; 4 Department of Medicine, Joondalup Health Campus, Joondalup, WA 6027, Australia; 5 Australian Institute of Tropical Health and Medicine, James Cook University, Townsville, QLD 4811, Australia; 6 Department of Vascular and Endovascular Surgery, Townsville University Hospital, Townsville, QLD 4814, Australia; 7 Queensland Research Centre for Peripheral Vascular Disease, College of Medicine and Dentistry, James Cook University, Townsville, QLD 4811, Australia; 8 Medical School, University of Western Australia, Perth, WA 6009, Australia; 9 Cardiometabolic Service, Department of Cardiology and Internal Medicine, Royal Perth Hospital, Perth, WA 6000, Australia; 10 Dobney Hypertension Centre, RPH Research Foundation, Perth, WA 6000, Australia; markus.schlaich@uwa.edu.au; 11 Department of Nephrology, Royal Perth Hospital, Perth, WA 6000, Australia

**Keywords:** lipoprotein(a), triglycerides, peripheral arterial disease, aortoiliac occlusive disease, stent restenosis, covered stent, endovascular revascularisation, Cox proportional hazards, biomarker

## Abstract

Background: Restenosis and occlusion remain important after aortoiliac stenting, and device choice does not fully explain late patency loss. Lipoprotein(a) [Lp(a)] is implicated in atherosclerotic progression and residual vascular risk. Objective: To determine whether baseline Lp(a) is associated with long-term restenosis or occlusion after aortoiliac stenting. Methods: This post hoc biomarker analysis of COBEST included 125 patients and 168 treated iliac arteries randomised to the Advanta V12 covered stent (Atrium Medical Corporation, Hudson, NH, USA) or bare-metal stents for symptomatic aortoiliac occlusive disease. The primary outcome was loss of primary patency, defined as restenosis or occlusion during 5-year follow-up. Cox proportional hazards modelling and cumulative failure curves were the main analyses. Results: In the Lp(a)-adjusted Cox model, covered stent use was associated with a lower hazard of restenosis or occlusion (HR, 0.55; 95% CI, 0.31–0.97; *p* = 0.039), whereas each 1 mg/dL higher Lp(a) concentration was associated with a higher hazard (HR, 1.08; 95% CI, 1.06–1.10; *p* < 0.001; equivalent to HR, 2.16; 95% CI, 1.79–2.59 per 10 mg/dL increase). In the biomarker-adjusted model, each 1 mg/dL higher Lp(a) concentration (HR, 1.05; 95% CI, 1.02–1.08; *p* < 0.001; HR, 1.63; 95% CI, 1.22–2.16 per 10 mg/dL increase) and each 1 mmol/L higher triglyceride concentration (HR, 1.89; 95% CI, 1.34–2.68; *p* < 0.001) remained associated with restenosis or occlusion. Conclusions: Elevated Lp(a) and, to a lesser extent, triglycerides were independently associated with long-term aortoiliac stent failure after adjustment for stent type.

## 1. Introduction

Aortoiliac occlusive disease is an important manifestation of peripheral arterial disease and reflects advanced systemic atherosclerosis. It is associated with lifestyle-limiting claudication, impaired functional capacity, chronic limb-threatening ischemia, and increased cardiovascular risk [1,2,3,4]. Although endovascular treatment is now widely used for symptomatic aortoiliac disease, long-term restenosis and occlusion remain important limitations, particularly in complex lesions and high-risk patients [1,2,3,4].

Lipoprotein(a) [Lp(a)] is a genetically determined LDL-like particle in which apolipoprotein B-100 is covalently bound to apolipoprotein(a). Lp(a) carries oxidized phospholipids, promotes endothelial dysfunction, may impair fibrinolysis, and is associated with systemic atherosclerotic cardiovascular disease and peripheral artery disease [5,6,7].

Homocysteine has been associated with endothelial dysfunction, oxidative stress, thrombosis, and peripheral artery disease [8]. These pathways are relevant to restenosis and occlusion after stent implantation, where endothelial injury, platelet activation, inflammation, smooth-muscle-cell proliferation, and extracellular matrix deposition contribute to late luminal loss.

Endovascular revascularization is an established treatment strategy for symptomatic aortoiliac occlusive disease, particularly in patients with functionally limiting symptoms or limb-threatening ischemia. Stent design may influence durability. Covered stents can exclude plaque and damaged endothelium from the arterial lumen, theoretically reducing neointimal ingrowth and late luminal loss compared with bare-metal stents [9,10].

The Covered Versus Balloon Expandable Stent Trial (COBEST) was a landmark multicenter randomised trial comparing covered balloon-expandable stents with bare-metal stents in aortoiliac occlusive disease [11]. The original 18-month report showed better freedom from binary restenosis with covered stents, especially in TASC C and D lesions [11]. Five-year follow-up subsequently confirmed more durable patency and fewer target-limb revascularizations in the covered-stent group [12].

Triglyceride-rich lipoproteins and remnant particles are increasingly recognized as contributors to residual inflammatory and atherothrombotic risk [13,14]. The present post hoc biomarker analysis was designed to determine whether baseline Lp(a) is associated with long-term restenosis or occlusion after aortoiliac stenting; whether this association persists after adjustment for stent type and related biomarkers; and whether biomarker-based risk information adds to device-related assessment of long-term patency.

## 2. Methods

### 2.1. Study Design, Ethical Approval, and Population

This study was a post hoc biomarker analysis of COBEST, a prospective, multicenter, randomised controlled trial comparing the Advanta V12 balloon-expandable covered stent (Atrium Medical Corporation, Hudson, NH, USA) with commercially available bare-metal stents in patients with symptomatic aortoiliac occlusive disease. The parent trial was conducted across eight major Australian centres between January 2006 and December 2008 by participating vascular specialists, including vascular surgeons and interventional radiologists. The present biomarker-focused analysis was undertaken within the established clinical follow-up and patency-outcome framework of the original trial to examine whether baseline Lp(a) was associated with long-term restenosis or occlusion after aortoiliac stenting, and whether stent type remained associated with patency after accounting for Lp(a) and related biomarkers. The trial design, eligibility criteria, procedural methods, and 18-month outcomes have been reported previously [11]. Five-year durability outcomes were subsequently collected through annual follow-up according to each institution’s protocol and have also been published [12].

The institutional review boards and human research ethics committees at the participating hospitals approved the parent trial protocol, and written informed consent was obtained from all participants before enrolment, as reported in the original COBEST publication [11]. The present analysis used de-identified trial data and was conducted within the established clinical follow-up and patency-outcome framework of COBEST. Reporting was aligned with the STROBE statement [15].

### 2.2. Eligibility Criteria

Eligible participants were men or women aged 18 years or older with symptomatic aortoiliac occlusive disease and TASC B, C, or D lesions, including haemodynamically significant dissections and recurrent stenosis after angioplasty. Patients were excluded if they had a life expectancy of less than 12 months, uncontrolled hypertension, TASC A lesions, pregnancy, prior enrolment or previous aortoiliac procedures, extensive common femoral artery disease or multiple groin procedures, contraindication to aspirin or clopidogrel, occluded superficial and profunda femoral arteries, inability to provide informed consent, or expected non-compliance with protocol requirements [11].

### 2.3. Therapy and Devices

Patients were randomised in a 1:1 ratio to receive either the Advanta V12 balloon-expandable covered stent (Atrium Medical Corporation, Hudson, NH, USA) or a commercially available bare-metal stent. The bare-metal stent type was selected at the discretion of the interventionist, provided the device had Australian Therapeutic Goods Administration approval. Stents were sized perioperatively, with covered and bare-metal stents generally oversized by 1 mm beyond the calculated diameter of the treated iliac artery. All patients received aspirin 100 to 150 mg daily indefinitely and clopidogrel 75 mg daily for at least 1 month after intervention, with a 300 mg loading dose of clopidogrel given during or after the procedure [11].

### 2.4. Study Procedures and Imaging Follow-Up

Arterial access, lesion crossing, angiographic assessment, and stent deployment were performed according to the parent trial protocol [11]. Digital subtraction angiography was performed to assess and confirm duplex ultrasound or computed tomography angiography findings. The patency status of ipsilateral femoral, popliteal, and infrapopliteal arteries was documented.

Clinical assessment, ankle–brachial index measurement, and aortoiliac duplex ultrasound surveillance were performed at 1, 6, 12, and 18 months after the index procedure, with annual follow-up thereafter according to each participating institution’s protocol for the five-year durability analysis [11,12]. If duplex ultrasound was technically limited, inconclusive, or clinically discordant, computed tomography angiography or digital subtraction angiography was performed using a prespecified protocol [11]. Imaging was performed as part of structured vascular follow-up using institutional vascular laboratory and radiology protocols. Formal interobserver or intraobserver repeatability testing of imaging measurements was not undertaken within the original trial dataset.

### 2.5. Biochemistry and Biomarker Assessment

Biochemical analyses were performed in hospital or clinic chemistry laboratories using standard local laboratory methods and reagents. Fasting venous blood samples were obtained at baseline. The assessed variables included full blood count, fasting lipid profile, glucose, renal and liver parameters, high-sensitivity C-reactive protein, glycated haemoglobin, homocysteine, total cholesterol, LDL cholesterol, triglycerides, and Lp(a). HDL cholesterol was not available in the analytic dataset and is therefore not reported. LDL cholesterol was calculated using the Friedewald formula. Diabetes mellitus was defined according to World Health Organization criteria as fasting blood glucose ≥ 7.0 mmol/L [16].

Total fasting serum homocysteine was measured from baseline blood samples using high-performance liquid chromatography, as described by Jacobsen et al. [17]. Because serum homocysteine concentrations may be approximately 10% to 30% higher than plasma concentrations, results were interpreted using the local serum reference range [17,18]. The local laboratory reference range was 0 to 15 micromol/L; therefore, homocysteine values > 15 micromol/L were considered elevated.

Lp(a) concentration was measured at the time of the original study in the hospital chemistry laboratory using the locally available Incstar assay (Incstar Corporation, Stillwater, MN, USA) according to the manufacturer’s instructions. This automated immunoprecipitation assay used a monospecific goat antibody directed against Lp(a). All Lp(a) values were reported in mg/dL, and these original mass units were retained for all analyses [5,19]. No conversion from mg/dL to nmol/L was performed because Lp(a) particle mass varies according to apolipoprotein(a) isoform size, and contemporary guidance advises against the use of a universal mass-to-molar conversion factor [5,19].

Lp(a) was analysed primarily as a continuous variable. For categorical analyses and adjusted cumulative failure curves, high Lp(a) was defined as >50 mg/dL, and values ≤ 50 mg/dL were used as the reference category. This threshold was consistent with the original dataset definition, prior epidemiological studies, and contemporary consensus guidance for increased Lp(a)-associated cardiovascular risk [5].

### 2.6. Outcome and Imaging Follow-Up

The primary outcome was loss of primary patency, defined as restenosis or occlusion of the treated aortoiliac segment during 5-year follow-up. Restenosis was defined as greater than 50% stenosis of the treated segment, assessed by duplex ultrasound, computed tomography angiography, digital subtraction angiography, or quantitative visual angiography. Occlusion was defined as complete loss of patency of the treated segment. Lesions within 5 mm of the proximal or distal stent margin were considered part of the treated segment, consistent with the 5-year durability analysis [12].

### 2.7. Statistical Analysis

The treated iliac artery was the unit of analysis because the primary outcome was vessel-level loss of primary patency, defined as restenosis or occlusion of the treated aortoiliac segment during follow-up. Patients who underwent bilateral iliac intervention could therefore contribute more than one treated artery. To account for within-patient correlation, Cox proportional hazards models used robust sandwich variance estimators clustered at the patient level, as described by Lin and Wei [20].

Baseline characteristics were summarized overall and according to stent type and Lp(a) category. Continuous variables are presented as mean ± SD or median with interquartile range, as appropriate, and categorical variables as number and percentage. Bare-metal stent was used as the reference category in all stent comparisons. Lp(a) was analyzed primarily as a continuous variable; for categorical analyses and graphical presentation, elevated Lp(a) was defined as >50 mg/dL.

Exploratory and supportive analyses were performed to examine the consistency of the biomarker signal across complementary methods. Univariable logistic regression with 1000 bootstrap replications was used as a fixed-time screening analysis of restenosis or occlusion at 5 years and is presented in the Supplementary Material to avoid over-emphasizing broad candidate-variable screening [21,22,23]. Receiver operating characteristic analysis, Spearman correlation analysis with false-discovery-rate adjustment, and LASSO variable selection were also interpreted as exploratory and supportive rather than as primary inferential analyses [24,25,26].

The main hypothesis was evaluated using Cox proportional hazards regression. Follow-up time was measured from the index procedure to restenosis, occlusion, or censoring. Tied event times were handled using the Efron approximation [27]. The principal Cox model included stent type and Lp(a). A biomarker-adjusted Cox model additionally included triglycerides, LDL cholesterol, and homocysteine. Results are reported as hazard ratios with 95% confidence intervals and two-sided p values. Covariate selection for the adjusted Cox models was guided by biological relevance to the Lp(a) hypothesis and by the number of available outcome events: during follow-up, 43 of 168 treated arteries (25.6%) developed restenosis or occlusion, corresponding to approximately 21.5 events per covariate for the 2-covariate Lp(a)-adjusted model (Model 1) and approximately 8.6 events per covariate for the 5-covariate biomarker-adjusted model (Model 2), using the conventional benchmark of approximately 10 events per covariate; the latter falls marginally below this threshold, and Model 2 was therefore treated as a secondary, more exploratory analysis. The covariates added in Model 2 (triglycerides, LDL cholesterol, and homocysteine) were restricted to lipid- and thiol-pathway biomarkers with direct biological relevance to Lp(a)-related atherothrombosis. Other baseline factors that differed between stent groups or Lp(a) categories, including smoking history, hypertension, and TASC classification, were examined descriptively and in univariable Cox analyses (Section 3.2) and in exploratory LASSO-based variable selection (Section 3.5) rather than entered into the adjusted models, to limit the risk of overfitting given the limited number of events. Data on lipid-lowering therapy (e.g., statin use) were not captured in the original COBEST database and could not be evaluated as a covariate or potential confounder; this is acknowledged as a limitation (Section 5).

Kaplan–Meier and cumulative failure curves were used to visualize the probability of restenosis or occlusion over time according to Lp(a) category and stent type. Cox-adjusted cumulative failure curves were generated to display the association between Lp(a) category and restenosis or occlusion after adjustment for stent type and relevant biomarkers. The proportional hazards assumption was assessed using scaled Schoenfeld residuals and global testing, and overall model fit was assessed using Cox–Snell residuals.

Multivariable fractional polynomial Cox modelling was used as a complementary time-to-event approach to retain continuous predictor information and allow flexible functional forms where these were supported by the data [28]. All statistical tests were two-sided. Findings were interpreted according to effect size, precision, consistency across Cox-based analyses, graphical time-to-event patterns, and biological plausibility rather than *p* values alone. Because this was a post hoc exploratory biomarker analysis, results were interpreted as prognostic associations rather than evidence of causal biomarker effects. Analyses were performed using Stata version 19 (StataCorp LLC, College Station, TX, USA).

## 3. Results

### 3.1. Clinical Presentation, Study Population, and Baseline Characteristics

The analysis included 125 patients contributing 168 treated iliac arteries. Forty-three patients underwent bilateral iliac intervention and therefore contributed two treated arteries each, accounting for the difference between the patient and artery totals; all Cox models were fitted at the artery level and used robust sandwich variance estimators clustered on patient to account for this within-patient correlation (Section 2.7). Covered balloon-expandable stents were used in 83 arteries and bare-metal stents in 85 arteries. At baseline, all patients had symptomatic aortoiliac occlusive disease. In the original COBEST cohort, symptoms were recorded using the Rutherford–Becker classification [11]. Intermittent claudication, corresponding to Rutherford–Becker categories 1–3, was present in 98 of 125 patients (78.4%): 5 patients (4.0%) had category 1 symptoms, 36 (28.8%) had category 2 symptoms, and 57 (45.6%) had category 3 symptoms. Rest pain, corresponding to category 4, was present in 23 patients (18.4%). Minor tissue loss or ulceration, corresponding to category 5, was present in 4 patients (3.2%). Rutherford–Becker category 6 disease, including major tissue loss or gangrene, was not separately reported in the original baseline table. In the present treated-artery dataset, critical limb ischemia was recorded in 33 of 168 treated arteries (19.6%).

Baseline clinical, biochemical, anatomical, and procedural characteristics according to stent type are summarised in Table 1. Hypertension, smoking history, diabetes mellitus, coronary artery disease, and pre-intervention antiplatelet use were common, reflecting a high-risk atherosclerotic population. TASC C/D lesions and critical limb ischaemia were more frequent among arteries treated with covered stents, consistent with the use of covered stents in more complex aortoiliac disease. Formal comparison between stent groups showed statistically significant differences for hypertension, TASC classification, and critical limb ischaemia (all *p* < 0.05), whereas age, sex, race, Lp(a), other biochemical measures, and the remaining clinical risk factors did not differ significantly between groups.

### 3.2. Characteristics According to Lp(a) Category

Baseline and outcome characteristics according to Lp(a) category are shown in Table 2. Overall, 42 treated arteries were in the elevated Lp(a) group, defined as Lp(a) > 50 mg/dL, and 126 were in the lower Lp(a) group, defined as Lp(a) ≤ 50 mg/dL. Treated arteries in the elevated Lp(a) group were from younger patients and were associated with higher total cholesterol, LDL cholesterol, triglyceride, and homocysteine concentrations. The distribution of TASC C/D lesions was broadly similar between Lp(a) groups, suggesting that the excess risk associated with elevated Lp(a) was not explained by measured anatomical complexity alone. The proportion of Caucasian patients was slightly lower in the elevated Lp(a) group (90.5% vs. 99.2%; Fisher’s exact *p* = 0.014); this modest baseline imbalance is distinct from, and numerically much less pronounced than, the strong univariable association between Caucasian race and a lower hazard of restenosis or occlusion shown in Table 2 (HR, 0.17; *p* = 0.001).

Restenosis or occlusion occurred in 31 of 42 treated arteries in the elevated Lp(a) group compared with 12 of 126 treated arteries in the lower Lp(a) group. In univariable Cox analysis, Lp(a) > 50 mg/dL was associated with a markedly higher hazard of restenosis or occlusion.

Procedural, anatomical, stent-related, and postprocedural characteristics according to Lp(a) category are shown in Table 3. Covered stent use was associated with a lower hazard of restenosis or occlusion, whereas absence of clinical improvement was more frequent in the elevated Lp(a) group and was associated with a higher hazard of subsequent restenosis or occlusion. The STROBE checklist and the original COBEST inclusion and exclusion criteria are provided in Tables S1 and S2. Additional descriptive analyses stratified by homocysteine and triglyceride category are provided in Tables S3 and S4, respectively.

### 3.3. Cumulative Failure According to Lp(a) Category

**Figure 1:**
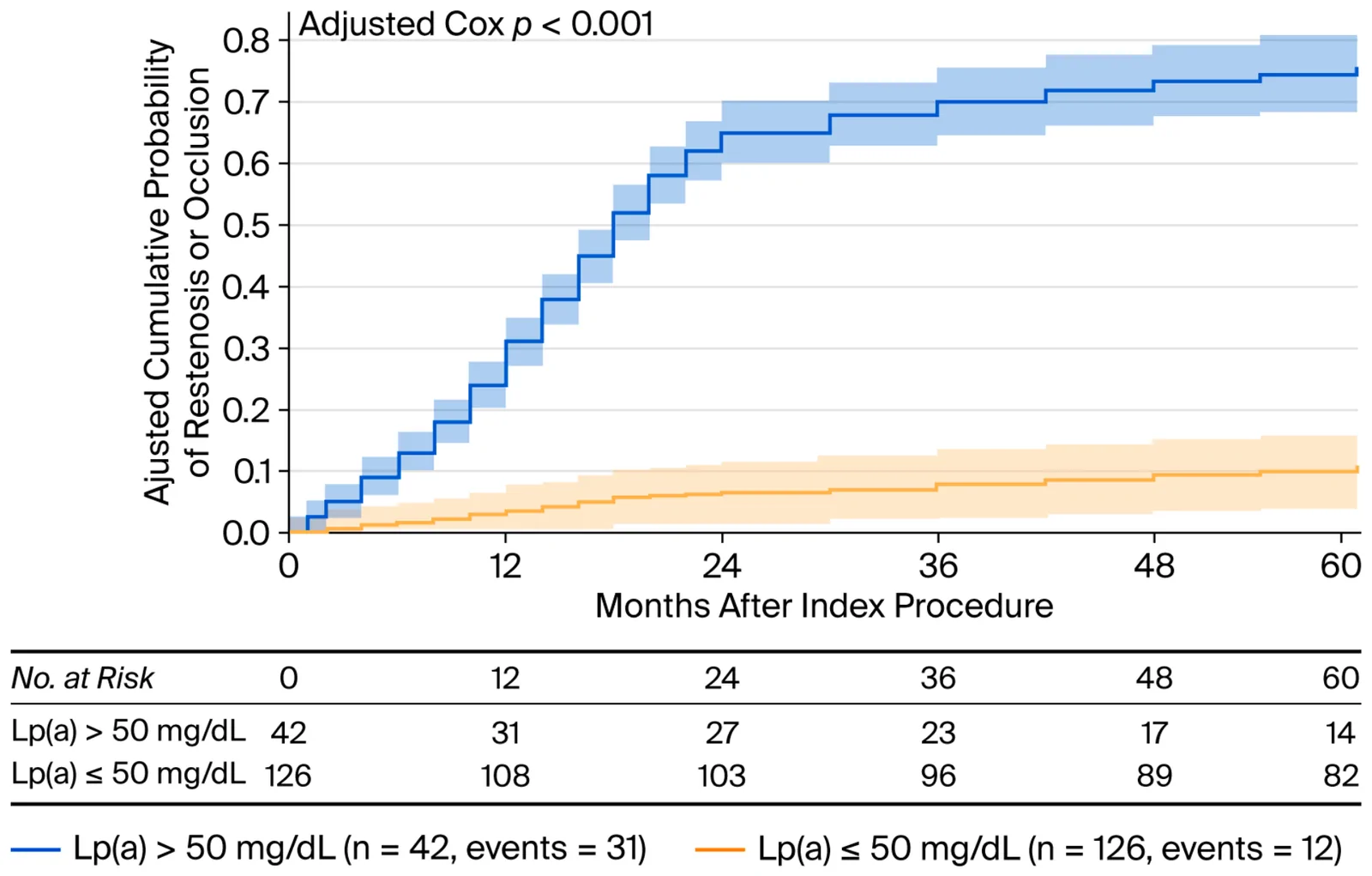
Biomarker-adjusted cumulative probability of restenosis or occlusion according to Lp(a) category. Biomarker-adjusted cumulative failure curves show the probability of restenosis or occlusion during 5-year follow-up according to baseline Lp(a) category. The blue curve represents treated arteries in patients with Lp(a) > 50 mg/dL (*n* = 42; events = 31), and the orange curve represents treated arteries in patients with Lp(a) ≤ 50 mg/dL (*n* = 126; events = 12). Shaded bands represent 95% confidence intervals. Curves were adjusted for stent type, triglycerides, LDL cholesterol, and homocysteine. The displayed *p* value was derived from the adjusted Cox proportional hazards model. A number-at-risk table is shown below the plot. Abbreviations: CI, confidence interval; LDL, low-density lipoprotein; Lp(a), lipoprotein(a).

Cumulative failure curves demonstrated a substantially higher probability of restenosis or occlusion among treated arteries from patients with Lp(a) > 50 mg/dL compared with those with Lp(a) ≤ 50 mg/dL. This separation persisted after adjustment for stent type, triglycerides, LDL cholesterol, and homocysteine, supporting an association between elevated Lp(a) and long-term loss of primary patency (Figure 1). Additional adjusted cumulative failure analysis according to stent type is provided in Figure S1.

### 3.4. Discrimination Analysis

**Figure 2:**
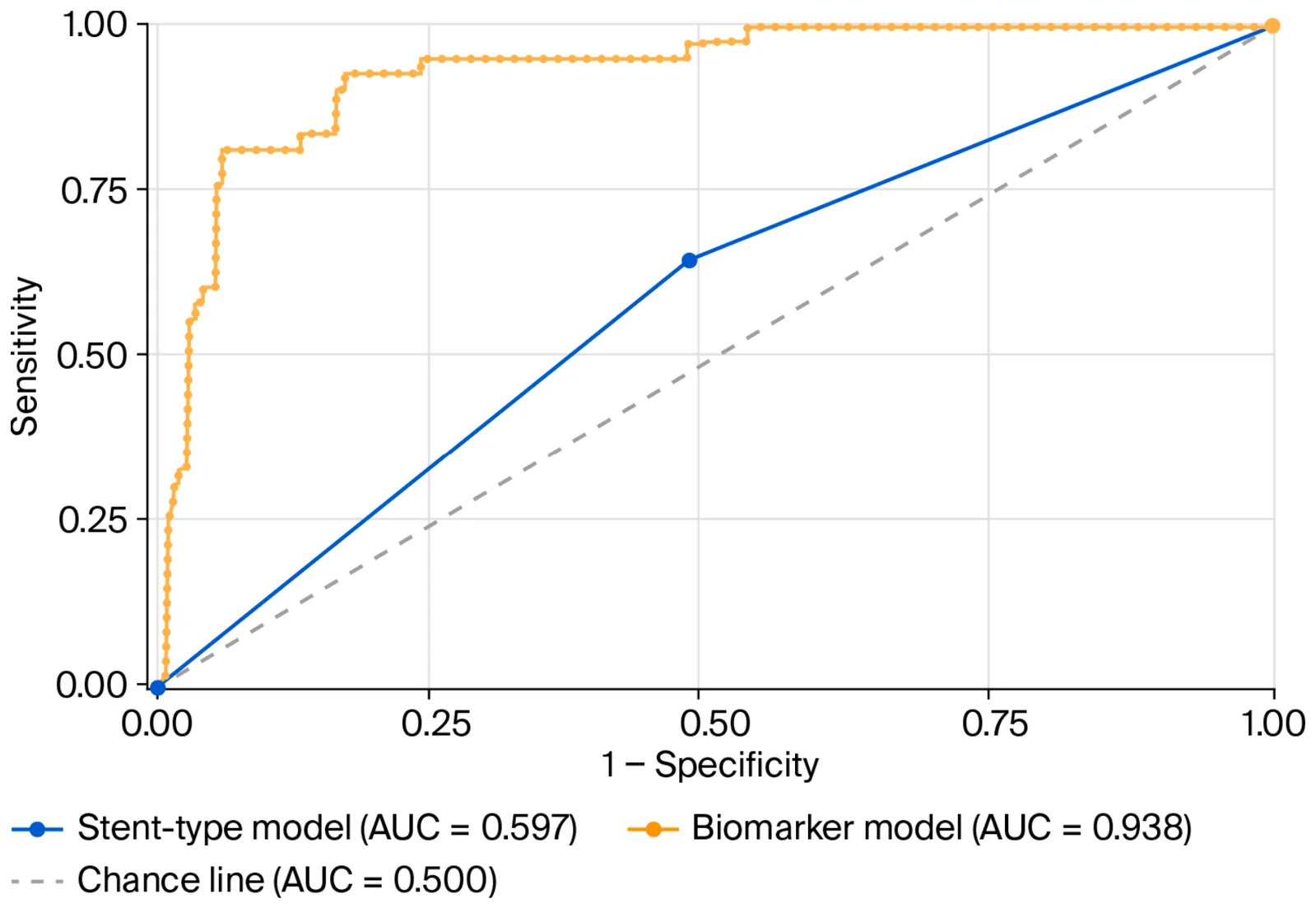
Receiver operating characteristic curves for stent-type and biomarker models. Receiver operating characteristic curves compare fixed-time discrimination for restenosis or occlusion at 5 years. The orange curve represents the biomarker model, which included Lp(a), triglycerides, LDL cholesterol, and homocysteine (AUC = 0.938). The blue curve represents the stent-type model comparing covered versus bare-metal stents (AUC = 0.597). The grey dashed diagonal line represents the chance line (AUC = 0.500). These analyses were interpreted descriptively as supportive evidence of model discrimination and not as a validated clinical prediction tool. Abbreviations: AUC, area under the receiver operating characteristic curve; LDL, low-density lipoprotein; Lp(a), lipoprotein(a); ROC, receiver operating characteristic.

Receiver operating characteristic analysis was performed as a supportive fixed-time discrimination analysis for restenosis or occlusion. The biomarker model showed stronger discrimination than the stent-type model, consistent with the hypothesis that patient-level biomarker biology contributed additional prognostic information beyond device type alone (Figure 2). These findings were interpreted descriptively and not as evidence of a validated clinical prediction model.

### 3.5. BIC-Based LASSO Variable Selection

**Figure 3:**
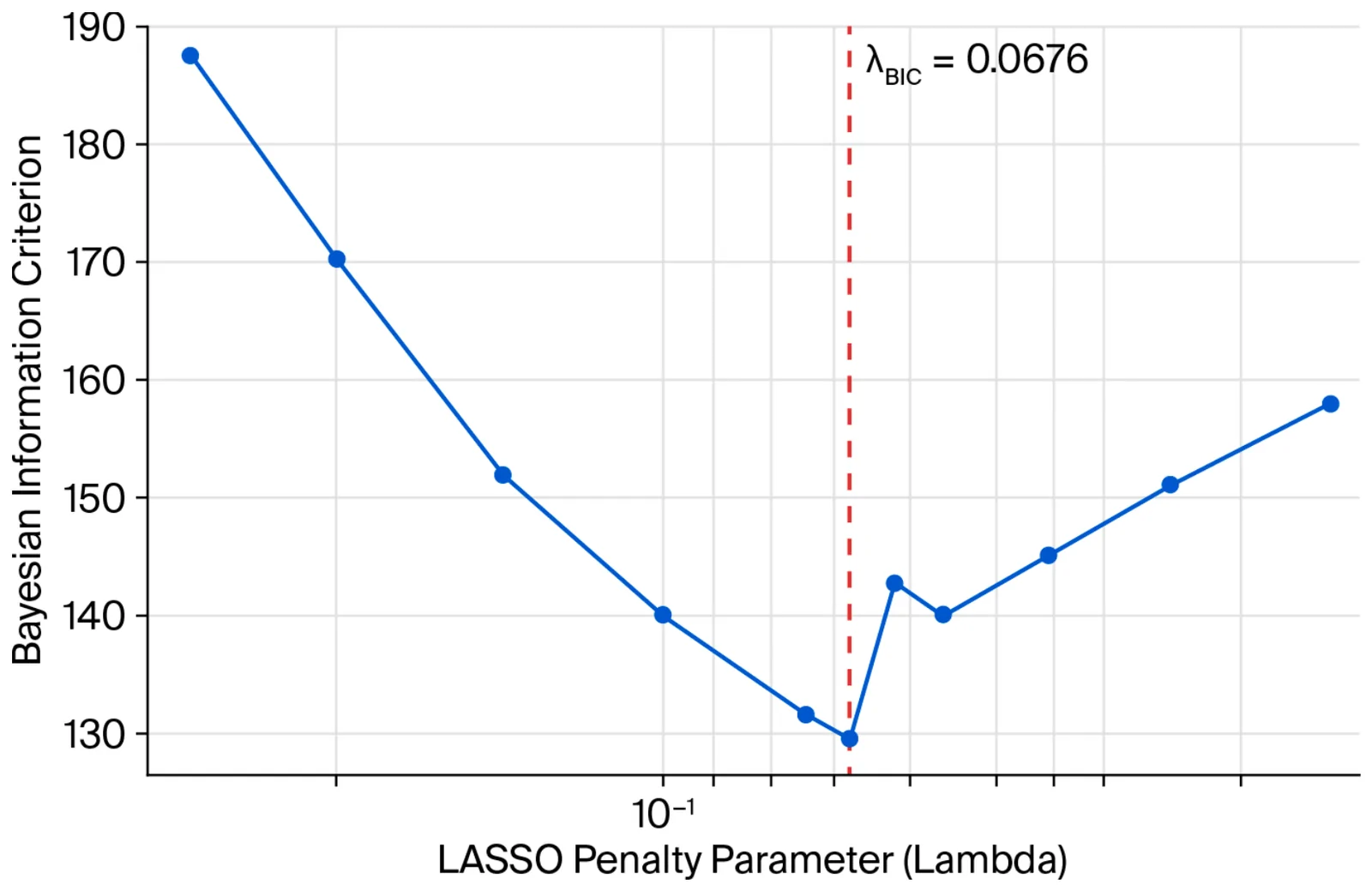
BIC-based LASSO penalty selection for restenosis or occlusion. The Bayesian information criterion (BIC) was plotted across candidate values of the LASSO penalty parameter, λ, to identify the most parsimonious penalised logistic regression model. Lower BIC values indicate better model fit after accounting for model complexity. The dark red dashed vertical line marks the BIC-selected penalty value, λ = 0.0676 (Table S6), which corresponded to the lowest BIC and retained five non-zero predictors: Lp(a), stent type, homocysteine, triglycerides, and LDL cholesterol. This analysis was used as supportive model-selection evidence and was not considered the primary inferential analysis. Abbreviations: BIC, Bayesian information criterion; LASSO, least absolute shrinkage and selection operator; LDL, low-density lipoprotein; Lp(a), lipoprotein(a).

**Figure 4:**
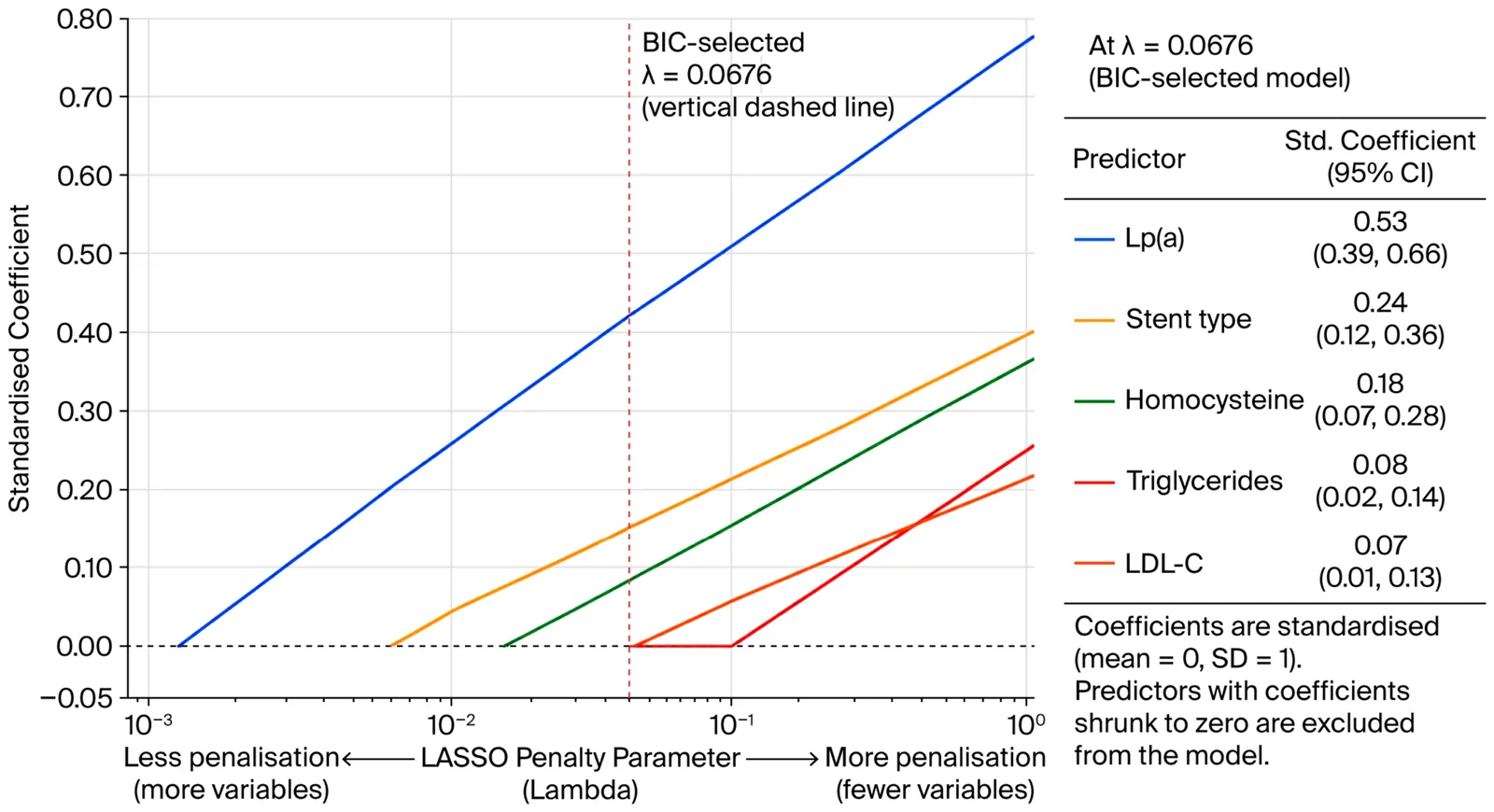
LASSO coefficient path plot for candidate predictors of restenosis or occlusion. The plot shows standardised coefficient paths from LASSO penalised logistic regression for candidate predictors of restenosis or occlusion. The x-axis represents the LASSO penalty parameter, λ, on a logarithmic scale, and the y-axis shows the corresponding standardised regression coefficient for each retained predictor. The dark red dashed vertical line identifies the Bayesian information criterion-selected penalty value, λ = 0.0676, which represents the model that best balanced model fit and parsimony. At this selected penalty, five predictors retained non-zero coefficients: Lp(a), stent type, homocysteine, triglycerides, and LDL-C. Predictors with coefficients shrunk to zero were excluded from the penalised model. The right-hand panel summarises the selected predictors and their standardised coefficients with 95% confidence intervals. This analysis was used as supportive variable-selection evidence and was not considered the primary inferential analysis. Abbreviations: BIC, Bayesian information criterion; CI, confidence interval; LASSO, least absolute shrinkage and selection operator; LDL-C, low-density lipoprotein cholesterol; Lp(a), lipoprotein(a).

BIC-based LASSO selection was used as a supportive model-selection approach. The selected penalty parameter retained five predictors: Lp(a), stent type, homocysteine, triglycerides, and LDL cholesterol. The BIC-based penalty selection plot is shown in Figure 3, and the LASSO coefficient path is shown in Figure 4. These analyses supported the stability of the biomarker signal, particularly the contribution of Lp(a), but were interpreted as supportive rather than primary inferential analyses. Detailed LASSO-selected coefficients and BIC penalty selection values are provided in Tables S5 and S6, respectively.

### 3.6. Cox Proportional Hazards Models

The primary time-to-event analyses are presented in Table 4. In the Lp(a)-adjusted Cox model, covered stent use was associated with a lower hazard of restenosis or occlusion compared with bare-metal stenting (HR, 0.55; 95% CI, 0.31–0.97; *p* = 0.039). Increasing Lp(a) was associated with a higher hazard of restenosis or occlusion after adjustment for stent type (HR, 1.08; 95% CI, 1.06–1.10; equivalent to HR, 2.16; 95% CI, 1.79–2.59 per 10 mg/dL increase; *p* < 0.001).

**Figure 5:**
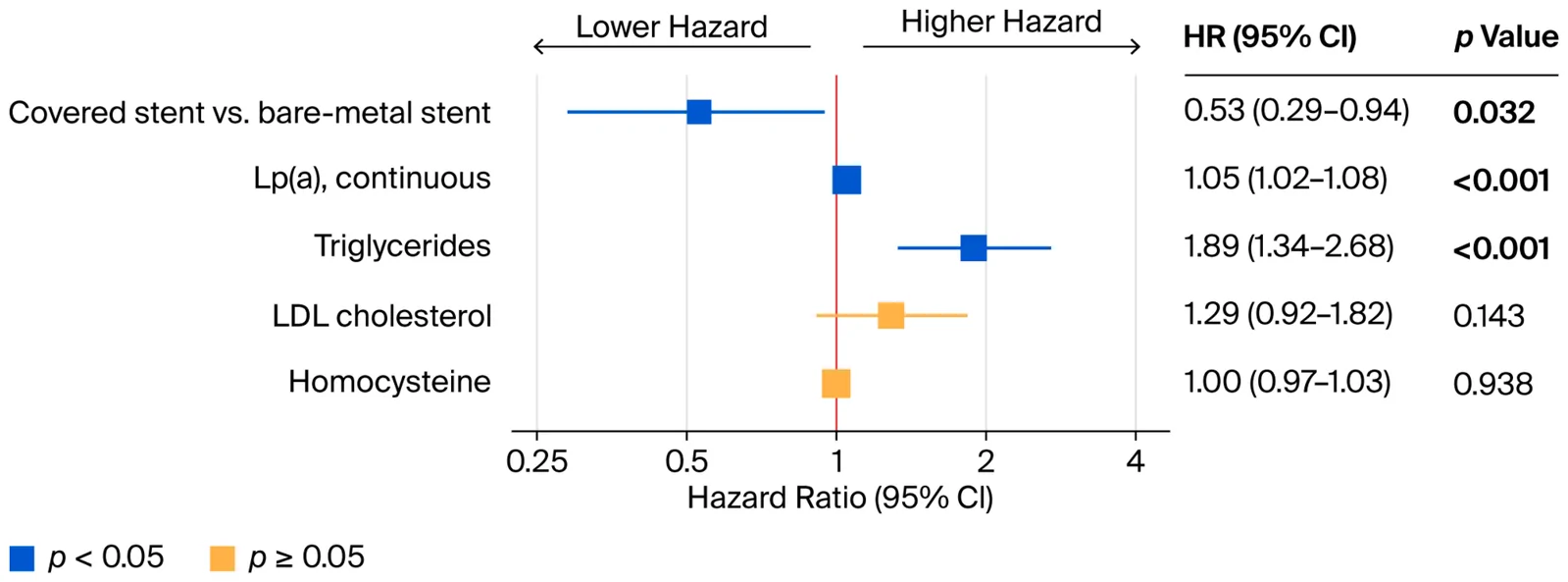
Biomarker-adjusted Cox proportional hazards model for restenosis or occlusion. Forest plot of the biomarker-adjusted Cox proportional hazards model for time to restenosis or occlusion during follow-up. This figure corresponds to Model 2 in Table 4. Bare-metal stent was the reference category. Continuous predictor hazard ratios are reported per 1 mg/dL increase in Lp(a), per 1 mmol/L increase in triglycerides and LDL cholesterol, and per 1 μmol/L increase in homocysteine. Hazard ratios less than 1 indicate a lower hazard of restenosis or occlusion. Abbreviations: CI, confidence interval; LDL, low-density lipoprotein; Lp(a), lipoprotein(a).

In the biomarker-adjusted Cox model, covered stent use remained associated with a lower hazard of restenosis or occlusion (HR, 0.53; 95% CI, 0.29–0.94; *p* = 0.032). Lp(a) also remained associated with restenosis or occlusion after adjustment for triglycerides, LDL cholesterol, homocysteine, and stent type (HR, 1.05; 95% CI, 1.02–1.08; equivalent to HR, 1.63; 95% CI, 1.22–2.16 per 10 mg/dL increase; *p* < 0.001). Triglycerides were also associated with a higher hazard of restenosis or occlusion in the biomarker-adjusted model (HR, 1.89; 95% CI, 1.34–2.68; *p* < 0.001), whereas LDL cholesterol and homocysteine were not independently associated with the outcome after adjustment. The biomarker-adjusted Cox estimates are displayed graphically in Figure 5.

### 3.7. Exploratory, Fractional Polynomial, and Diagnostic Analyses

The full univariable candidate-predictor forest plot with bootstrap validation was moved to the Supplementary Material to avoid over-emphasising broad exploratory screening. This analysis is provided in Figure S2 and was interpreted as descriptive only. The Spearman correlation analysis, including false discovery rate adjustment, is shown in Figure S3 and was used to examine monotonic associations and potential collinearity among candidate predictors.

As a complementary time-to-event analysis, fractional polynomial Cox modelling retained Lp(a), triglycerides, and stent type in the final model. Lp(a) was modelled using a square-root transformation, while triglycerides were retained as a linear term. Covered stent use remained associated with a lower hazard of restenosis or occlusion compared with bare-metal stenting. The final fractional polynomial Cox model is provided in Table S7.

Model diagnostics supported the adequacy of the Cox-based modelling approach. Overall model fit, assessed using Cox–Snell residuals, was adequate, with a non-significant test of equality (Grønnesby–Borgan *p* = 0.56; Figure S4). Scaled Schoenfeld residuals and the Grambsch–Therneau test showed no evidence of a time-dependent covariate effect for Lp(a) (χ^2^ = 1.21, *p* = 0.27), triglycerides (χ^2^ = 0.84, *p* = 0.36), or stent type (χ^2^ = 1.32, *p* = 0.25); the global test was similarly non-significant (χ^2^ = 2.23, *p* = 0.53), indicating no detectable violation of the proportional hazards assumption (Figure S5).

## 4. Discussion

In this post hoc, observational biomarker analysis of COBEST, baseline Lp(a) was associated with long-term restenosis or occlusion after endovascular treatment of symptomatic aortoiliac occlusive disease. As this was a non-randomised, hypothesis-generating secondary analysis rather than a study designed a priori to test the Lp(a) hypothesis, the findings below are presented as associations and should not be interpreted as establishing a causal effect of Lp(a) on stent failure. The association was evident in cumulative failure analyses and remained present in Cox proportional hazards models after adjustment for stent type and related biomarkers. Elevated Lp(a), defined as >50 mg/dL, identified a subgroup with substantially higher risk of late loss of primary patency. These findings suggest that long-term aortoiliac stent failure may be influenced not only by device-related factors and lesion complexity but also by patient-level atherothrombotic biology.

The principal finding is that Lp(a) was associated with restenosis or occlusion independently of stent type. In the Lp(a)-adjusted Cox model, higher Lp(a) was associated with increased hazard of loss of primary patency, while covered stent use was associated with lower hazard compared with bare-metal stenting. This pattern persisted in the biomarker-adjusted model, in which Lp(a) and triglycerides remained associated with restenosis or occlusion, whereas LDL cholesterol and homocysteine were not independently associated after adjustment. These results support a continuum of biological risk rather than a simple device-only explanation for late stent failure.

Although the point estimate for triglycerides (HR, 1.89 per 1 mmol/L) exceeded that for Lp(a) (HR, 1.05 per 1 mg/dL) in the biomarker-adjusted model, these hazard ratios are not directly comparable because they are expressed per unit of very different scales. When expressed per standard deviation of the cohort’s own distribution (Lp(a), 15.7 mg/dL; triglycerides, 0.8 mmol/L), the standardised hazard ratio for Lp(a) (approximately 2.15 per SD) exceeds that for triglycerides (approximately 1.69 per SD), indicating a comparatively stronger graded association with restenosis or occlusion across the observed range of Lp(a) than the raw per-unit hazard ratio suggests. Similarly, when expressed per 10 mg/dL increase in Lp(a), the hazard ratio for Lp(a) was 1.63 (95% CI, 1.22–2.16) in the biomarker-adjusted model, and the categorical comparison of Lp(a) > 50 mg/dL versus ≤50 mg/dL was associated with a markedly higher univariable hazard (HR, 9.63; 95% CI, 5.00–18.57; Table 2), consistent across all three scalings of the same underlying association. Lp(a) and triglycerides also differ in clinical actionability: triglyceride concentration is modifiable by lifestyle, fibrates, and n-3 fatty acids and remains an important, guideline-endorsed cardiovascular risk factor in its own right [18,19], a point developed further below. Lp(a), by contrast, is under near-exclusive genetic control and is not meaningfully altered by diet, exercise, or conventional lipid-lowering therapy, so it identifies a subgroup of patients whose elevated vascular risk would otherwise go unrecognised by standard lipid testing; this, together with emerging Lp(a)-targeted therapies discussed below, motivates the emphasis placed on Lp(a) in the present analysis despite its smaller unadjusted per-unit effect size.

These findings extend the original COBEST observations. The parent trial showed that covered balloon-expandable stents improved freedom from binary restenosis compared with bare-metal stents, particularly in TASC C and D lesions [11]. The 5-year durability analysis subsequently confirmed more durable patency and fewer target-limb revascularisations in the covered-stent group [12]. The present analysis adds a biomarker dimension to those device-based findings by showing that elevated Lp(a) identifies treated iliac arteries at increased risk of long-term failure despite revascularisation. The persistence of the Lp(a) association after adjustment for stent type indicates that Lp(a) is not simply a marker of device allocation or procedural strategy.

The device-related findings also remain clinically relevant. Covered stent use was associated with lower hazard of restenosis or occlusion compared with bare-metal stenting, even after adjustment for Lp(a) and other biomarkers. This suggests that stent design continues to matter in long-term aortoiliac durability. Conversely, the persistence of the Lp(a) signal after adjustment for stent type suggests that biological risk is not fully overcome by device choice. These findings support an integrated interpretation in which local mechanics, lesion morphology, stent design, and systemic vascular biology all contribute to late patency outcomes.

The broader literature supports the relevance of Lp(a) to systemic and polyvascular atherosclerotic disease. Lp(a) has been associated with stenotic atherosclerosis, lesion severity, and the number of affected vascular beds [29]. Other contemporary studies emphasise that the anatomic distribution of peripheral atherosclerosis and residual biomarker risk may identify different dimensions of cardiovascular risk [30,31]. Our findings extend these observations from disease burden and cardiovascular outcomes to the durability of peripheral endovascular reconstruction.

Contemporary trials and registry studies also highlight the ongoing uncertainty around optimal aortoiliac device strategy. The DISCOVER trial reported no significant difference in 2-year primary patency between covered and bare-metal stents in the common iliac artery, whereas COBEST demonstrated a more durable benefit of covered stents in complex aortoiliac disease [32]. Covered endovascular reconstruction of the aortic bifurcation has further expanded treatment options for complex aortoiliac occlusive disease [33,34]. Whether Lp(a) predicts durability after these contemporary approaches remains an important question for future investigation.

The biological plausibility of Lp(a) as a determinant of restenosis or occlusion is strong. Lp(a) combines LDL-like cholesterol carriage with apolipoprotein(a)-mediated proinflammatory and prothrombotic effects. It is also a major carrier of oxidised phospholipids, which may promote endothelial dysfunction, vascular inflammation, platelet activation, impaired fibrinolysis, and smooth-muscle-cell proliferation [5,6,29,35]. These pathways are directly relevant to the vascular response after stent implantation, where endothelial injury, inflammation, neointimal hyperplasia, and thrombosis contribute to restenosis and occlusion. A high-Lp(a) environment may therefore amplify the biological processes that drive late luminal loss.

Triglycerides were also associated with restenosis or occlusion in the biomarker-adjusted Cox model. This finding is consistent with the concept that triglyceride-rich lipoproteins and remnant particles contribute to residual atherosclerotic risk beyond LDL cholesterol alone [13,14,36]. In the present analysis, triglycerides remained associated with loss of patency after adjustment for Lp(a), LDL cholesterol, homocysteine, and stent type. By contrast, LDL cholesterol and homocysteine did not remain independently associated with restenosis or occlusion in the biomarker-adjusted Cox model. These findings suggest that Lp(a), the biology of triglyceride-rich lipoprotein, and stent type captured the most consistent risk domains in the available dataset.

The ROC and LASSO analyses should be interpreted as supportive rather than primary evidence. The ROC analysis suggested stronger discrimination for the biomarker model than for the stent-type model alone, supporting the concept that circulating biomarkers may add prognostic information beyond device selection. BIC-based LASSO selection also retained Lp(a), stent type, homocysteine, triglycerides, and LDL cholesterol, with Lp(a) contributing prominently. However, these analyses were exploratory and are not sufficient to establish a validated prediction model. The primary inference remains based on the cumulative failure curves and Cox proportional hazards models.

These results have potential clinical implications. Measurement of Lp(a) is increasingly recommended in cardiovascular risk assessment, particularly because Lp(a) is largely genetically determined, relatively stable over time, and associated with atherosclerotic cardiovascular disease [5,6]. In patients undergoing aortoiliac stenting, elevated Lp(a) may identify individuals who warrant closer duplex surveillance, aggressive optimisation of modifiable risk factors, and careful long-term follow-up. At present, the findings should not be used to mandate a specific device strategy or treatment pathway, but they do support the inclusion of Lp(a) in future studies on the durability of peripheral revascularisation.

The findings may also be relevant to the emerging therapeutic landscape. PCSK9 inhibitors can modestly lower Lp(a), and more specific Lp(a)-lowering therapies, including antisense oligonucleotide and small interfering RNA approaches, are under investigation [37]. Whether lowering Lp(a) improves peripheral stent patency, reduces target-limb revascularisation, or improves limb outcomes remains unknown. Patients with PAD and elevated Lp(a) may represent an important, enriched population for future trials evaluating whether biomarker-targeted therapy can modify the durability of revascularisation.

This study has several strengths. It used long-term follow-up from a well-characterised randomised aortoiliac intervention cohort, included both covered and bare-metal stents, assessed a clinically meaningful patency outcome, and accounted for clustering when patients contributed bilateral treated arteries. The convergence of cumulative failure curves and Cox proportional hazards models strengthens the interpretation that elevated Lp(a) is associated with late restenosis or occlusion.

## 5. Limitations

This study has several limitations. It was a post hoc biomarker analysis and was not designed to prove causality. The sample size was modest, and the number of restenosis or occlusion events limited the complexity of multivariable modelling. Although clustering by patient was addressed statistically, residual confounding remains possible, including from unmeasured factors such as lipid-lowering therapy (e.g., statin use), which was not captured in the original COBEST database and could not be evaluated as a covariate.

Imaging follow-up was performed according to the original COBEST surveillance framework and institutional protocols; no formal interobserver or intraobserver repeatability testing of imaging measurements was undertaken. Lp(a) was measured at baseline, and serial changes in Lp(a) or other biomarkers were not available. The findings require external validation in contemporary cohorts using current aortoiliac devices, standardised imaging protocols, and prespecified biomarker analysis plans.

## 6. Conclusions

Elevated Lp(a) was associated with long-term restenosis or occlusion after aortoiliac stenting, independently of stent type and related biomarkers. Covered stent use remained associated with lower hazard of late failure, while triglycerides also contributed to biomarker-adjusted risk. These findings suggest that the long-term durability of aortoiliac stents reflects both device-related and patient-level biological factors. Future studies should determine whether Lp(a)-guided surveillance, intensified prevention, or targeted Lp(a)-lowering therapy can improve patency and limb outcomes after peripheral endovascular revascularisation.

## 7. Perspectives

Competency in medical knowledge: In patients undergoing aortoiliac stenting, elevated Lp(a) may identify increased risk of late restenosis or occlusion beyond device type alone.

Translational outlook: Prospective studies are required to validate whether Lp(a)-guided surveillance, intensified secondary prevention, or emerging Lp(a)-lowering therapies can improve long-term patency and limb outcomes after peripheral endovascular revascularisation.

## Figures and Tables

**Table 1:** Baseline clinical, biochemical, anatomical, and procedural characteristics according to stent type.

Characteristic	Covered Stent, (*n* = 83)	Bare-Metal Stent, (*n* = 85)	Total, (*n* = 168)	*p* Value
Demographic characteristics				
Age—yr	66.3 ± 10.9	66.3 ± 10.2	66.3 ± 10.5	0.951
Male sex—No. (%)	50 (60.2)	53 (62.4)	103 (61.3)	0.779
Caucasian race—No. (%)	82 (98.8)	81 (95.3)	163 (97.0)	0.368
Clinical risk factors				
Hypertension—No. (%)	49 (59.0)	63 (74.1)	112 (66.7)	0.038
Diabetes mellitus—No. (%)	22 (26.5)	23 (27.1)	45 (26.8)	0.936
Smoking history—No. (%)	51 (61.5)	53 (62.4)	104 (61.9)	0.904
Chronic renal failure—No. (%)	3 (3.6)	2 (2.4)	5 (3.0)	0.680
Coronary artery disease—No. (%)	32 (38.6)	29 (34.1)	61 (36.3)	0.550
Chronic obstructive airways disease—No. (%)	6 (7.2)	8 (9.4)	14 (8.3)	0.609
Hypercoagulation history—No. (%)	6 (7.2)	3 (3.5)	9 (5.4)	0.326
Pre-intervention antiplatelet therapy—No. (%)	73 (88.0)	80 (94.1)	153 (91.1)	0.161
Biochemical characteristics				
Total cholesterol—mmol/L	5.1 ± 1.8	5.3 ± 1.6	5.2 ± 1.7	0.489
Lp(a)—mg/dL	36.0 ± 15.0	38.8 ± 16.4	37.4 ± 15.7	0.243
Triglycerides—mmol/L	1.4 ± 0.9	1.5 ± 0.8	1.5 ± 0.8	0.495
LDL cholesterol—mmol/L	1.8 ± 1.1	2.0 ± 1.1	1.9 ± 1.1	0.272
Homocysteine—μmol/L	13.5 ± 9.8	15.2 ± 11.2	14.4 ± 10.5	0.284
Anatomical characteristics				
Preoperative ABI	0.5 ± 0.2	0.5 ± 0.2	0.5 ± 0.2	0.799
TASC B lesion—No. (%)	41 (49.4)	58 (68.2)	99 (58.9)	0.025
TASC C/D lesion—No. (%)	42 (50.6)	27 (31.8)	69 (41.1)	0.025
Critical limb ischaemia—No. (%)	22 (26.5)	11 (12.9)	33 (19.6)	0.027

Note: Data are presented as mean ± SD or number (%). *p* values compare the covered-stent and bare-metal-stent groups using Welch’s *t*-test for continuous variables or the chi-square test (Fisher’s exact test where an expected cell count was <5) for categorical variables; no adjustment for multiple comparisons was made, and these baseline comparisons are descriptive. Abbreviations: ABI, ankle–brachial index; LDL, low-density lipoprotein; Lp(a), lipoprotein(a); TASC, Trans-Atlantic Inter-Society Consensus.

**Table 2:** Clinical, biochemical, anatomical, and outcome characteristics according to Lp(a) category.

Characteristic	Lp(a) ≤ 50 mg/dL, *n* = 126	Lp(a) > 50 mg/dL, *n* = 42	Total, *n* = 168	Cox HR (95% CI) †	*p* Value
Demographic characteristics					
Age—year	67.2 ± 10.4	61.5 ± 10.4	66.3 ± 10.5	0.80 (0.59–1.08) ‡	0.142
Male sex—No. (%)	76 (60.3)	27 (64.3)	103 (61.3)	1.02 (0.53–1.98)	0.953
Caucasian race—No. (%)	125 (99.2)	38 (90.5)	163 (97.0)	0.17 (0.06–0.49)	0.001
Stent treatment					
Bare-metal stent—No. (%)	61 (48.4)	24 (57.1)	85 (50.6)	Reference	—
Covered stent—No. (%)	65 (51.6)	18 (42.9)	83 (49.4)	0.49 (0.25–0.94)	0.033
Clinical characteristics					
Hypertension—No. (%)	87 (69.0)	25 (59.5)	112 (66.7)	0.69 (0.35–1.34)	0.273
Diabetes mellitus—No. (%)	35 (27.8)	10 (23.8)	45 (26.8)	0.91 (0.43–1.92)	0.806
Smoking history—No. (%)	75 (59.5)	29 (69.0)	104 (61.9)	2.61 (1.23–5.54)	0.012
Coronary artery disease—No. (%)	52 (41.3)	9 (21.4)	61 (36.3)	0.61 (0.27–1.40)	0.246
Biochemical characteristics					
Total cholesterol—mmol/L	5.0 ± 1.8	5.9 ± 1.0	5.2 ± 1.7	1.15 (0.98–1.34) §	0.088
Triglycerides—mmol/L	1.3 ± 0.7	2.0 ± 0.9	1.5 ± 0.8	2.39 (1.82–3.14) §	<0.001
LDL cholesterol—mmol/L	1.4 ± 0.8	3.3 ± 0.5	1.9 ± 1.1	2.04 (1.56–2.68) §	<0.001
Homocysteine—μmol/L	11.0 ± 6.6	24.5 ± 13.3	14.4 ± 10.5	1.34 (1.21–1.49) ¶	<0.001
Anatomical characteristics					
Preoperative ABI	0.55 ± 0.18	0.51 ± 0.18	0.54 ± 0.18	1.13 (0.96–1.33) **	0.155
TASC C/D lesion—No./total No. (%)	51/126 (40.5)	18/42 (42.9)	69/168 (41.1)	1.20 (0.63–2.31)	0.583
Critical limb ischaemia—No. (%)	21 (16.7)	12 (28.6)	33 (19.6)	1.97 (0.99–3.90)	0.052
Five-year outcome					
Restenosis or occlusion—No. (%)	12 (9.5)	31 (73.8)	43 (25.6)	9.63 (5.00–18.57) ††	<0.001

Note: Data are presented as mean ± SD or number (%) unless otherwise indicated. † The Cox HR and *p* value in this column quantify the univariable association between each baseline characteristic and time to restenosis or occlusion across the whole cohort; they are not a test of whether that characteristic differs between the Lp(a) categories shown in the preceding columns. For example, the *p* value of 0.001 beside Caucasian race reflects the strength of the association between race and restenosis or occlusion (HR, 0.17), not the between-group difference in the proportion of Caucasian patients across Lp(a) categories (99.2% vs. 90.5%), which was smaller (Fisher’s exact *p* = 0.014). ‡ Hazard ratio per 10-year increase in age. § Hazard ratio per 1 mmol/L increase. ¶ Hazard ratio per 5 μmol/L increase in homocysteine. ** Hazard ratio per 0.1-unit lower ABI. †† Hazard ratio for Lp(a) > 50 mg/dL versus Lp(a) ≤ 50 mg/dL. Bare-metal stent was the reference category for stent comparisons. Abbreviations: ABI, ankle–brachial index; CI, confidence interval; LDL, low-density lipoprotein; Lp(a), lipoprotein(a); TASC, Trans-Atlantic Inter-Society Consensus.

**Table 3:** Procedural, anatomical, stent-related, and postprocedural characteristics according to Lp(a) category.

Characteristic	Lp(a) ≤ 50 mg/dL, *n* = 126	Lp(a) > 50 mg/dL, *n* = 42	Total, *n* = 168	Cox HR (95% CI) †	*p* Value
Stent treatment characteristics					
Bare-metal stent—No. (%)	61 (48.4)	24 (57.1)	85 (50.6)	Reference	-
Covered stent—No. (%)	65 (51.6)	18 (42.9)	83 (49.4)	0.49 (0.25–0.94)	0.033
Number of stents used	1.19 ± 0.40	1.07 ± 0.26	1.16 ± 0.37	0.49 (0.17–1.40) ‡	0.184
One stent used No./total No. (%)	100/124 (80.6)	39/42 (92.9)	139/166 (83.7)	Reference	-
Two stents used No./total No. (%)	24/124 (19.4)	3/42 (7.1)	27/166 (16.3)	0.50 (0.18–1.44)	0.200
Anatomical and haemodynamic characteristics					
Preoperative ABI	0.55 ± 0.18	0.51 ± 0.18	0.54 ± 0.18	1.13 (0.96–1.33) §	0.155
Preoperative run-off vessels	2.97 ± 0.95	2.93 ± 1.02	2.96 ± 0.97	0.95 (0.67–1.34) ‡	0.755
≤2 preoperative run-off vessels—No./total No. (%)	36/124 (29.0)	14/42 (33.3)	50/166 (30.1)	1.56 (0.78–3.12)	0.207
TASC B lesion—No./total No. (%)	75/126 (59.5)	24/42 (57.1)	99/168 (58.9)	Reference	-
TASC C/D lesion—No./total No. (%)	51/126 (40.5)	18/42 (42.9)	69/168 (41.1)	1.20 (0.63–2.31)	0.583
Critical limb ischaemia—No. (%)	21 (16.7)	12 (28.6)	33 (19.6)	1.97 (0.99–3.90)	0.052
Postprocedural clinical response					
Clinical improvement—No./total No. (%)	120/124 (96.8)	33/42 (78.6)	153/166 (92.2)	Reference	-
No clinical improvement—No./total No. (%)	4/124 (3.2)	9/42 (21.4)	13/166 (7.8)	4.62 (1.38–15.49)	0.013

Note: Data are presented as mean ± SD, number (%), or number/total number (%) unless otherwise indicated. † Cox hazard ratios are univariable estimates for time to restenosis or occlusion. ‡ Hazard ratio per one-unit increase. § Hazard ratio per 0.1-unit lower ABI. Bare-metal stent, one stent used, TASC B lesion, and clinical improvement were the reference categories where applicable. Abbreviations: ABI, ankle–brachial index; CI, confidence interval; Lp(a), lipoprotein(a); TASC, Trans-Atlantic Inter-Society Consensus.

**Table 4:** Cox proportional hazards models for restenosis or occlusion.

Model	Predictor	Hazard Ratio	95% CI	*p* Value
Model 1: Lp(a)-adjusted model	Covered vs. bare-metal stent	0.55	0.31–0.97	0.039
Model 1: Lp(a)-adjusted model	Lp(a), per 1 mg/dL increase	1.08	1.06–1.10	<0.001
Model 2: biomarker-adjusted model	Covered vs. bare-metal stent	0.53	0.29–0.94	0.032
Model 2: biomarker-adjusted model	Lp(a), per 1 mg/dL increase	1.05	1.02–1.08	<0.001
Model 2: biomarker-adjusted model	Triglycerides, per 1 mmol/L increase	1.89	1.34–2.68	<0.001
Model 2: biomarker-adjusted model	LDL cholesterol, per 1 mmol/L increase	1.29	0.92–1.82	0.143
Model 2: biomarker-adjusted model	Homocysteine, per 1 μmol/L increase	1.00	0.97–1.03	0.938

Note: Cox proportional hazards models were used to evaluate time to restenosis or occlusion during follow-up. Model 1 included stent type and Lp(a). Model 2 additionally included triglycerides, LDL cholesterol, and homocysteine. Bare-metal stent was the reference category. Continuous predictor hazard ratios are reported per 1 mg/dL increase in Lp(a), per 1 mmol/L increase in triglycerides and LDL cholesterol, and per 1 μmol/L increase in homocysteine. Hazard ratios less than 1 indicate a lower hazard of restenosis or occlusion. Because per-1-unit hazard ratios for Lp(a) are sensitive to the (arbitrary) unit chosen, equivalent hazard ratios per 10 mg/dL increase in Lp(a) were 2.16 (95% CI, 1.79–2.59) in Model 1 and 1.63 (95% CI, 1.22–2.16) in Model 2; the corresponding categorical hazard ratio for Lp(a) >50 mg/dL vs. ≤50 mg/dL is reported separately in Table 2. Abbreviations: CI, confidence interval; LDL, low-density lipoprotein; Lp(a), lipoprotein(a).

## Data Availability

The data underlying this post hoc analysis are not publicly available because of ethics, governance, and participant confidentiality restrictions. De-identified data may be made available from the corresponding author upon reasonable request and subject to institutional approval.

## Supplementary Materials

The following supporting information can be downloaded at: https://www.mdpi.com/article/10.3390/jcdd13100513/s1, Supplementary (Tables S1–S7 and Figures S1–S5), containing Table S1: STROBE checklist summary; Table S2: Original COBEST inclusion and exclusion criteria; Table S3: Selected characteristics according to homocysteine category; Table S4: Selected characteristics according to triglyceride category; Table S5: LASSO-selected predictors of restenosis or occlusion; Table S6: BIC-based LASSO penalty selection for restenosis or occlusion; Table S7: Final multivariable fractional polynomial Cox model for restenosis or occlusion; Figure S1: Adjusted cumulative probability of restenosis or occlusion according to stent type; Figure S2: Exploratory univariable screening and bootstrap validation for restenosis or occlusion at 5 years; Figure S3: Spearman rank correlation analysis of candidate predictors of restenosis or occlusion; Figure S4: Cox–Snell residual assessment of Cox model fit. The Nelson–Aalen cumulative hazard estimate of the Cox–Snell residuals is plotted against the Cox–Snell residual scale; Figure S5: Scaled Schoenfeld residual assessment of the proportional hazards assumption.
