# Supplementary material for: Lipoprotein(a) and Triglycerides as Independent Predictors of Long-Term Aortoiliac Stent Failure After Endovascular Revascularisation
Source: J Cardiovasc Dev Dis. 2026 Oct 11;13(10):513. doi: 10.3390/jcdd13100513 (PMC13705251; doi:10.3390/jcdd13100513)
Supplement: Supplementary file 1 [file jcdd-13-00513-s001.zip › jcdd-4567599-supplementary.pdf]

Journal of Cardiovascular Development and Disease

Supplementary Materials

Lipoprotein(a) and Triglycerides as Independent Predictors of Long-Term Aortoiliac Stent Failure After Endovascular Revascularisation

Bibombe Patrice Mwipatayi[1,*], Bernardette Jingfei Lee[2], Davina Dauda[3], James Evan Dodd[2], Mariah Theresa Mwipatayi[4], Jonathan Golledge[5,6,7], Trevor Anthony Mori[8], Gerald Francis Watts[8,9] and Markus Peter Schlaich[10,11]

Tables S1–S7 and Figures S1–S5

Supplementary Tables

Table S1. STROBE checklist summary.

STROBE item	Reporting element	Manuscript location
Title and abstract	Study design, objective, main exposure, outcome, and principal findings reported	Title; Abstract
Introduction: background	Scientific rationale and clinical context described	Introduction
Introduction: objectives	Study objective and hypothesis stated	End of Introduction
Study design	Post hoc biomarker analysis described	Methods
Setting	Source cohort and clinical setting described	Methods
Participants	Eligibility criteria and treated iliac artery as unit of analysis described	Methods
Variables	Exposure, outcome, covariates, procedural variables, and biomarkers defined	Methods
Data sources and measurement	Biomarker measurement and clinical data collection described	Methods
Bias	Post hoc design, residual confounding, and assay limitations acknowledged	Limitations
Study size	Number of treated iliac arteries and stent groups reported	Methods; Results; Table 1
Statistical methods	Logistic regression, bootstrap validation, ROC analysis, Spearman correlation, LASSO, Cox modelling, and fractional polynomial Cox modelling described	Statistical Analysis
Main results	Cox, LASSO, ROC, and fractional polynomial model findings reported	Results; Table 4; Figures 1–5; Tables S5–S7
Other analyses	Residual diagnostics and proportional hazards assessment reported	Results; Figures S4 and S5
Interpretation	Prognostic rather than causal interpretation stated	Discussion; Limitations
Ethics	Ethics approval and consent reported	Methods

Note: Summary of manuscript reporting according to the STROBE checklist. Abbreviations: STROBE, Strengthening the Reporting of Observational Studies in Epidemiology.

Table S2. Original COBEST inclusion and exclusion criteria.

Inclusion Criteria	Exclusion Criteria
Men and women aged ≥18 years	Life expectancy <12 months
Informed consent obtained	Uncontrolled hypertension
Evidence of TASC B, C, or D lesions	TASC A lesion
Haemodynamically significant dissections or recurrent stenosis after angioplasty	Pregnancy or women of childbearing potential not using effective contraception
	Prior enrolment in the trial or previous procedure performed at the aortoiliac level
	Extensive common femoral artery disease or multiple groin procedures
	Contraindication to aspirin or clopidogrel
	Occluded superficial femoral and profunda femoral arteries
	Mental condition or language barrier preventing informed consent
	Uncooperative attitude or potential non-compliance with protocol requirements

Note: Original inclusion and exclusion criteria for the Covered Versus Balloon Expandable Stent Trial. Abbreviations: TASC, Trans-Atlantic Inter-Society Consensus.

Table S3. Selected characteristics according to homocysteine category.

Characteristic	Homocysteine ≤15 µmol/L, n = 114	Homocysteine >15 µmol/L, n = 54	Total, n = 168	Cox HR (95% CI) †	p Value
**Demographic characteristics**					
Age—yr	65.6 ± 10.6	65.9 ± 10.8	66.3 ± 10.5	0.80 (0.59–1.08) ‡	0.142
Male sex—No. (%)	68 (59.6)	35 (64.8)	103 (61.3)	1.02 (0.53–1.98)	0.953
Caucasian race—No. (%)	111 (97.4)	52 (96.3)	163 (97.0)	0.17 (0.06–0.49)	0.001
**Stent treatment and clinical characteristics**					
Bare-metal stent—No. (%)	57 (50.0)	28 (51.9)	85 (50.6)	Reference	—
Covered stent—No. (%)	57 (50.0)	26 (48.1)	83 (49.4)	0.49 (0.25–0.94)	0.033
Smoking history—No. (%)	65 (57.0)	39 (72.2)	104 (61.9)	2.61 (1.23–5.54)	0.012
Coronary artery disease—No. (%)	44 (38.6)	17 (31.5)	61 (36.3)	0.61 (0.27–1.40)	0.246
Critical limb ischaemia—No. (%)	17 (14.9)	16 (29.6)	33 (19.6)	1.97 (0.99–3.90)	0.052
**Biochemical and anatomical characteristics**					
Total cholesterol—mmol/L	5.0 ± 1.8	5.6 ± 1.5	5.2 ± 1.7	1.15 (0.98–1.34) §	0.088
Lp(a) >50 mg/dL—No. (%)	14 (12.3)	28 (51.9)	42 (25.0)	9.63 (5.00–18.57)	<0.001
Triglycerides—mmol/L	1.3 ± 0.7	1.8 ± 1.0	1.5 ± 0.8	2.39 (1.82–3.14) §	<0.001
LDL cholesterol—mmol/L	1.6 ± 0.9	2.6 ± 1.2	1.9 ± 1.1	2.04 (1.56–2.68) §	<0.001
Preoperative ABI	0.55 ± 0.18	0.52 ± 0.18	0.54 ± 0.18	1.13 (0.96–1.33) ¶	0.155
TASC C/D lesion—No. (%)	42 (36.8)	27 (50.0)	69 (41.1)	1.20 (0.63–2.31)	0.583
**Outcome**					
Restenosis or occlusion—No. (%)	15 (13.2)	28 (51.9)	43 (25.6)	4.82 (2.48–9.37)	<0.001

Note: Data are presented as mean ± SD or number (%) unless otherwise indicated. Bare-metal stent was the reference category for stent comparisons. † Cox hazard ratio (HR) per one-unit increase in the characteristic unless otherwise specified; ‡ HR per 10-year increase in age; § HR per 1 mmol/L increase; ¶ HR per 0.1-unit lower ABI. Abbreviations: ABI, ankle–brachial index; CI, confidence interval; HR, hazard ratio; LDL, low-density lipoprotein; Lp(a), lipoprotein(a); TASC, Trans-Atlantic Inter-Society Consensus.

Table S4. Selected characteristics according to triglyceride category.

Characteristic	Triglycerides <2.0 mmol/L, n = 135	Triglycerides ≥2.0 mmol/L, n = 33	Total, n = 168	Cox HR (95% CI) †	p Value
**Demographic characteristics**					
Age—yr	66.1 ± 10.5	64.1 ± 11.2	66.3 ± 10.5	0.80 (0.59–1.08) ‡	0.142
Male sex—No. (%)	84 (62.2)	19 (57.6)	103 (61.3)	1.02 (0.53–1.98)	0.953
Caucasian race—No. (%)	133 (98.5)	30 (90.9)	163 (97.0)	0.17 (0.06–0.49)	0.001
**Stent treatment and clinical characteristics**					
Bare-metal stent—No. (%)	67 (49.6)	18 (54.5)	85 (50.6)	Reference	—
Covered stent—No. (%)	68 (50.4)	15 (45.5)	83 (49.4)	0.49 (0.25–0.94)	0.033
Smoking history—No. (%)	81 (60.0)	23 (69.7)	104 (61.9)	2.61 (1.23–5.54)	0.012
Coronary artery disease—No. (%)	56 (41.5)	5 (15.2)	61 (36.3)	0.61 (0.27–1.40)	0.246
Critical limb ischaemia—No. (%)	23 (17.0)	10 (30.3)	33 (19.6)	1.97 (0.99–3.90)	0.052
**Biochemical and anatomical characteristics**					
Total cholesterol—mmol/L	5.1 ± 1.8	5.5 ± 1.3	5.2 ± 1.7	1.15 (0.98–1.34) §	0.088
Lp(a) >50 mg/dL—No. (%)	23 (17.0)	19 (57.6)	42 (25.0)	9.63 (5.00–18.57)	<0.001
LDL cholesterol—mmol/L	1.7 ± 1.1	2.5 ± 1.0	1.9 ± 1.1	2.04 (1.56–2.68) §	<0.001
Homocysteine >15 µmol/L—No. (%)	34 (25.2)	20 (60.6)	54 (32.1)	4.82 (2.48–9.37)	<0.001
Preoperative ABI	0.55 ± 0.18	0.51 ± 0.18	0.54 ± 0.18	1.13 (0.96–1.33) ¶	0.155
TASC C/D lesion—No. (%)	50 (37.0)	19 (57.6)	69 (41.1)	1.20 (0.63–2.31)	0.583
**Outcome**					
Restenosis or occlusion—No. (%)	21 (15.6)	22 (66.7)	43 (25.6)	5.40 (3.10–9.42)	<0.001

Note: Data are presented as mean ± SD or number (%) unless otherwise indicated. Bare-metal stent was the reference category for stent comparisons. † Cox hazard ratio (HR) per one-unit increase in the characteristic unless otherwise specified; ‡ HR per 10-year increase in age; § HR per 1 mmol/L increase; ¶ HR per 0.1-unit lower ABI. Abbreviations: ABI, ankle–brachial index; CI, confidence interval; HR, hazard ratio; LDL, low-density lipoprotein; Lp(a), lipoprotein(a); TASC, Trans-Atlantic Inter-Society Consensus.

Table S5. LASSO-selected predictors of restenosis or occlusion.

Predictor	Predictor Domain	Standardised LASSO Coefficient	Rank	Selected Lambda	Cross-Fit Beta	Cross-Fit OR	95% CI	p Value
Lp(a)	Biomarker	0.78292	1	0.067623	0.091	1.10	1.04–1.15	0.001
Bare-metal stent use	Device/procedural	0.39791	2	0.067623	0.912	2.49	0.82–7.60	0.109
Homocysteine	Biomarker	0.37521	3	0.067623	0.093	1.10	1.02–1.18	0.013
Triglycerides	Biomarker	0.25012	4	0.067623	0.654	1.92	0.91–4.04	0.085
LDL cholesterol	Biomarker	0.22616	5	0.067623	0.089	1.09	0.43–2.78	0.852

Note: Predictors were selected using Bayesian information criterion-based LASSO logistic regression. Standardised LASSO coefficients indicate the relative contribution of each selected predictor within the penalised model. Cross-fit estimates provide post-selection odds ratios and confidence intervals and were interpreted alongside Cox proportional hazards and fractional polynomial Cox models. Lp(a) was the highest-ranked selected predictor. Abbreviations: BIC, Bayesian information criterion; CI, confidence interval; LASSO, least absolute shrinkage and selection operator; LDL, low-density lipoprotein; Lp(a), lipoprotein(a); OR, odds ratio.

Table S6. BIC-based LASSO penalty selection for restenosis or occlusion.

ID	Description	Lambda	No. of Non-Zero Coefficients	In-Sample Deviance Ratio	BIC
1	First lambda	0.272997	1	0.0331	187.5718
15	Lambda before selected value	0.074217	5	0.4466	131.7897
16*	Selected lambda	0.067623	5	0.4578	129.7204
17	Lambda after selected value	0.061616	8	0.4692	142.7177
18	Last lambda	0.056142	8	0.4836	140.0799

Note: The LASSO penalty parameter was selected using the Bayesian information criterion. The selected value was $\lambda = 0.067623$, corresponding to the lowest BIC and retaining five non-zero predictors. *Lambda selected by the Bayesian information criterion. Abbreviations: BIC, Bayesian information criterion; LASSO, least absolute shrinkage and selection operator.

Table S7. Final multivariable fractional polynomial Cox model for restenosis or occlusion.

Predictor	Functional Form	Beta Coefficient	Robust SE	z Statistic	Hazard Ratio	95% CI	p Value
Lp(a)	FP1: x^0.5	0.063	0.011	5.50	1.065	1.041–1.089	<0.001
Triglycerides	FP1: x^1	0.659	0.182	3.62	1.933	1.354–2.762	<0.001
Covered vs. bare-metal stent	Linear	−0.661	0.303	−2.18	0.516	0.285–0.935	0.029

Note: The final multivariable fractional polynomial Cox model retained Lp(a), triglycerides, and stent type. Fractional polynomial modelling preserved continuous-predictor information and allowed flexible functional forms where supported by the data. Bare-metal stent was the reference category. Hazard ratios less than 1 indicate a lower hazard of restenosis or occlusion. Abbreviations: CI, confidence interval; FP, fractional polynomial; HR, hazard ratio; Lp(a), lipoprotein(a); SE, standard error.

Supplementary Figures

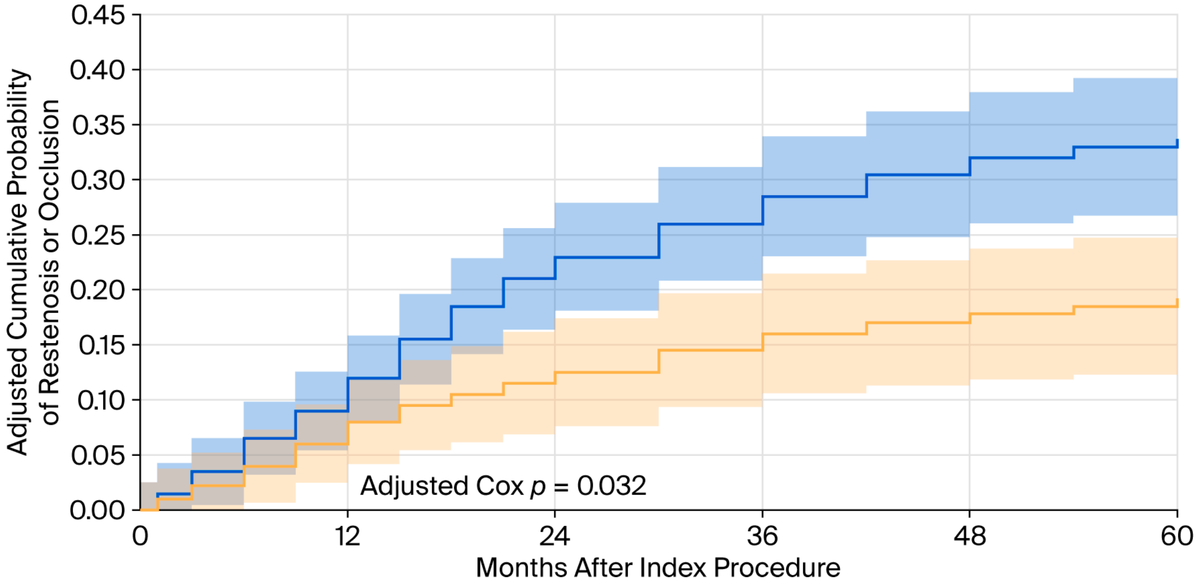

No. at risk	0	12	24	36	48	60
Bare-metal stent	85	75	68	59	51	44
Covered stent	83	77	71	64	57	50

— Bare-metal stent (n = 85, events = 28) — Covered stent (n = 83, events = 15)

Figure S1. Adjusted cumulative probability of restenosis or occlusion according to stent type. Adjusted cumulative failure curves show the probability of restenosis or occlusion during 5-year follow-up according to stent type. The blue curve represents bare-metal stents (n = 85; events = 28), and the orange curve represents covered stents (n = 83; events = 15). Shaded bands represent 95% confidence intervals. Curves were derived from the biomarker-adjusted Cox proportional hazards model, which included stent type, Lp(a), triglycerides, LDL cholesterol, and homocysteine. Bare-metal stent was the reference category; the displayed p value corresponds to the adjusted Cox comparison for covered versus bare-metal stents. The number-at-risk table is shown below the plot. Abbreviations: CI, confidence interval; LDL, low-density lipoprotein; Lp(a), lipoprotein(a).

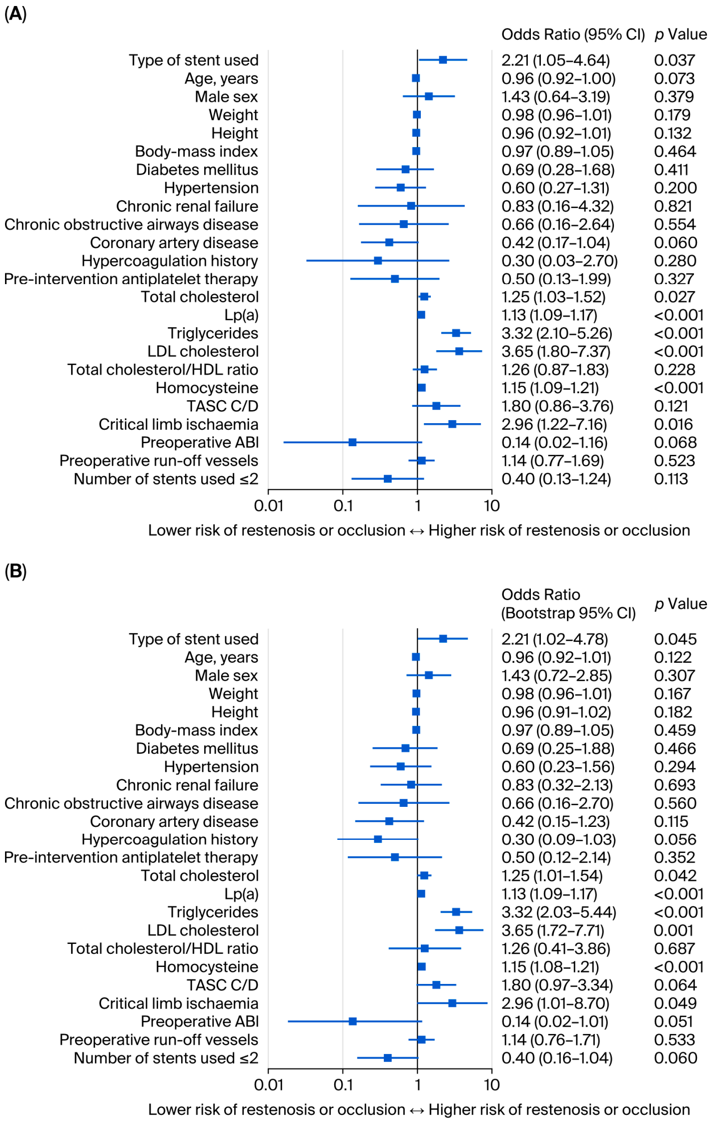

Figure S2. Exploratory univariable screening and bootstrap validation for restenosis or occlusion at 5 years. Panel A shows univariable odds ratios with robust 95% confidence intervals. Panel B shows bootstrap-validated odds ratios with bootstrap 95% confidence intervals. This analysis was exploratory and was not used as the primary inferential model. Abbreviations: ABI, ankle–brachial index; CI, confidence interval; LDL, low-density lipoprotein; Lp(a), lipoprotein(a); OR, odds ratio; TASC, Trans-Atlantic Inter-Society Consensus.

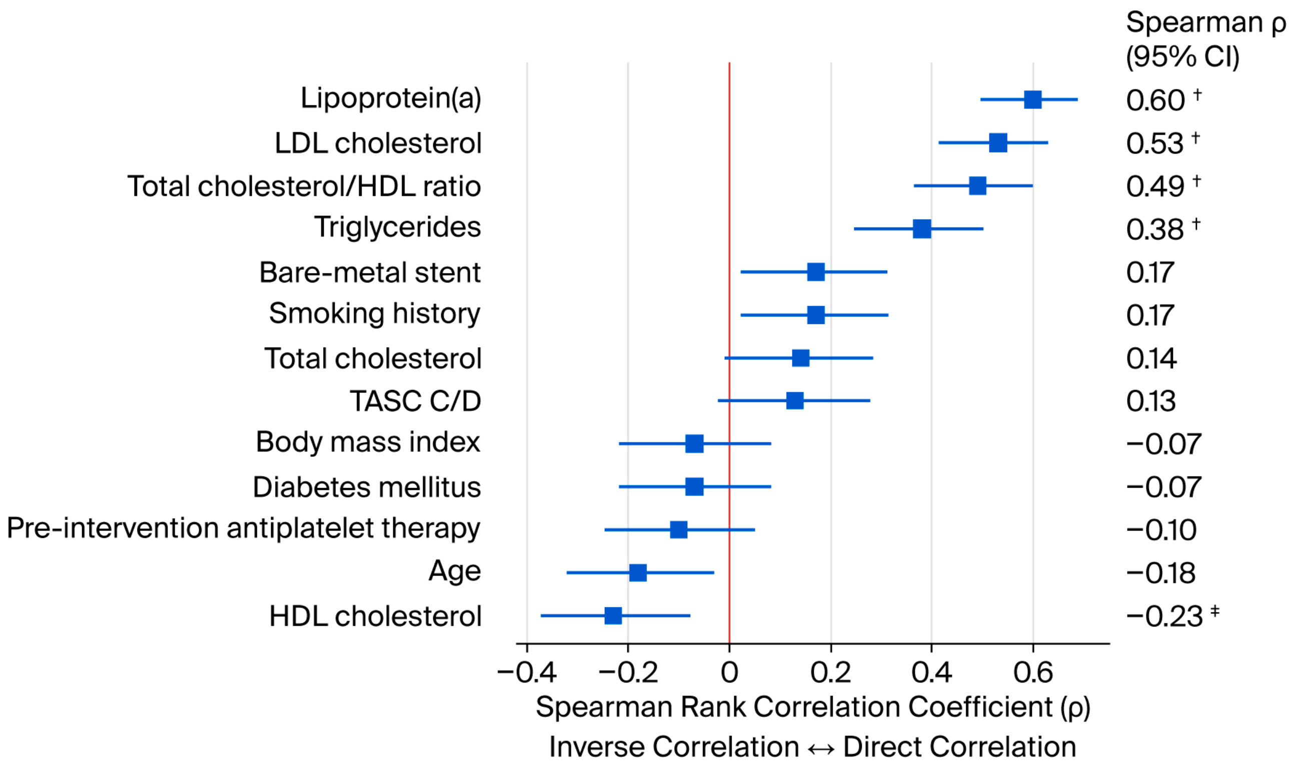

Figure S3. Spearman rank correlation analysis of candidate predictors of restenosis or occlusion. Squares represent Spearman rank correlation coefficients (ϱ), and horizontal lines represent 95% confidence intervals. The dark red vertical line indicates ϱ = 0, corresponding to no monotonic association; values to the right indicate direct correlation and values to the left indicate inverse correlation. † indicates statistical significance after Benjamini–Hochberg false discovery rate adjustment ($q < 0.05$). ‡ Analyses involving HDL cholesterol included 159 observations after exclusion of 9 implausible values. Abbreviations: CI, confidence interval; FDR, false discovery rate; HDL, high-density lipoprotein; LDL, low-density lipoprotein; TASC, Trans-Atlantic Inter-Society Consensus.

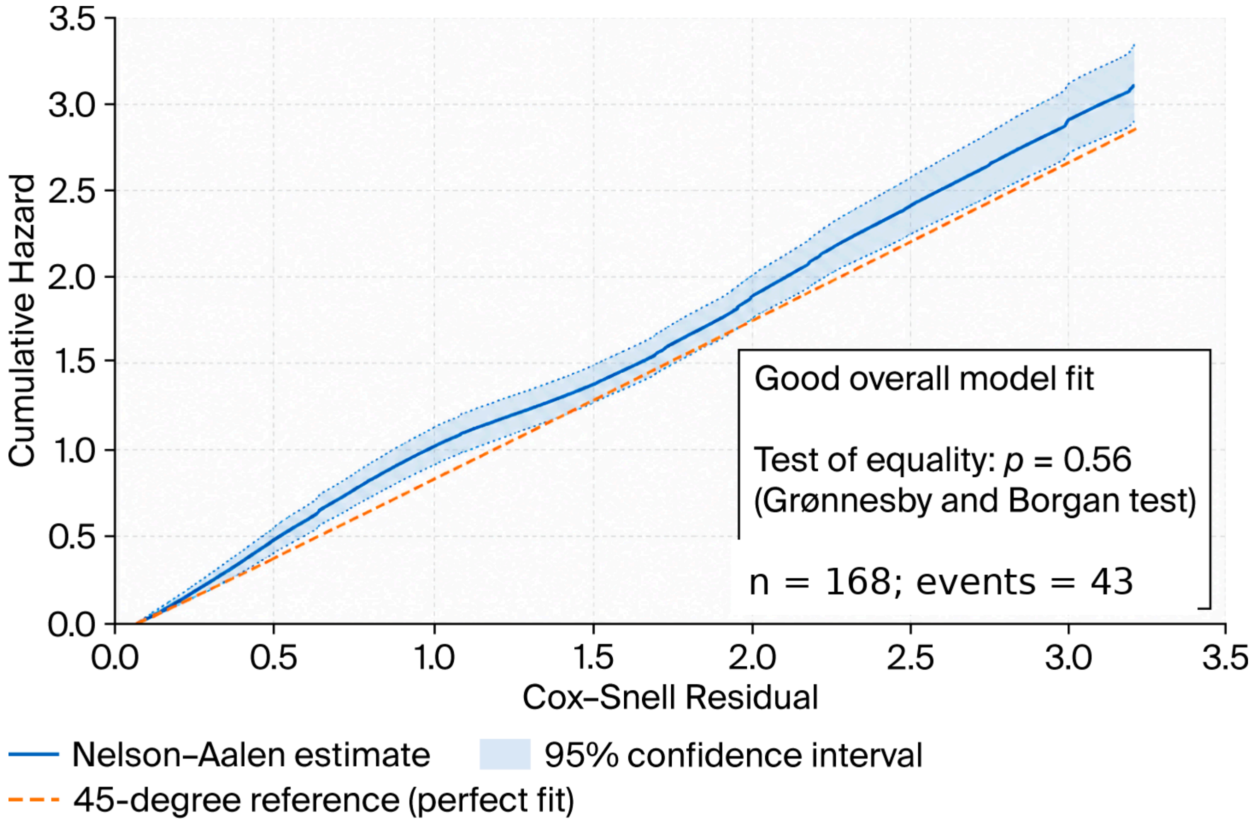

Figure S4. Cox–Snell residual assessment of Cox model fit. The Nelson–Aalen cumulative hazard estimate of the Cox–Snell residuals is plotted against the Cox–Snell residual scale. The orange dashed 45-degree reference line represents the expected pattern under adequate Cox model fit; closer agreement between the Nelson–Aalen estimate and the reference line indicates better overall calibration of the time-to-event model. The shaded band represents the 95% confidence interval around the Nelson–Aalen estimate. The plot shows close alignment with the reference line, supporting acceptable overall model fit for the Cox proportional hazards model. Abbreviations: CI, confidence interval.

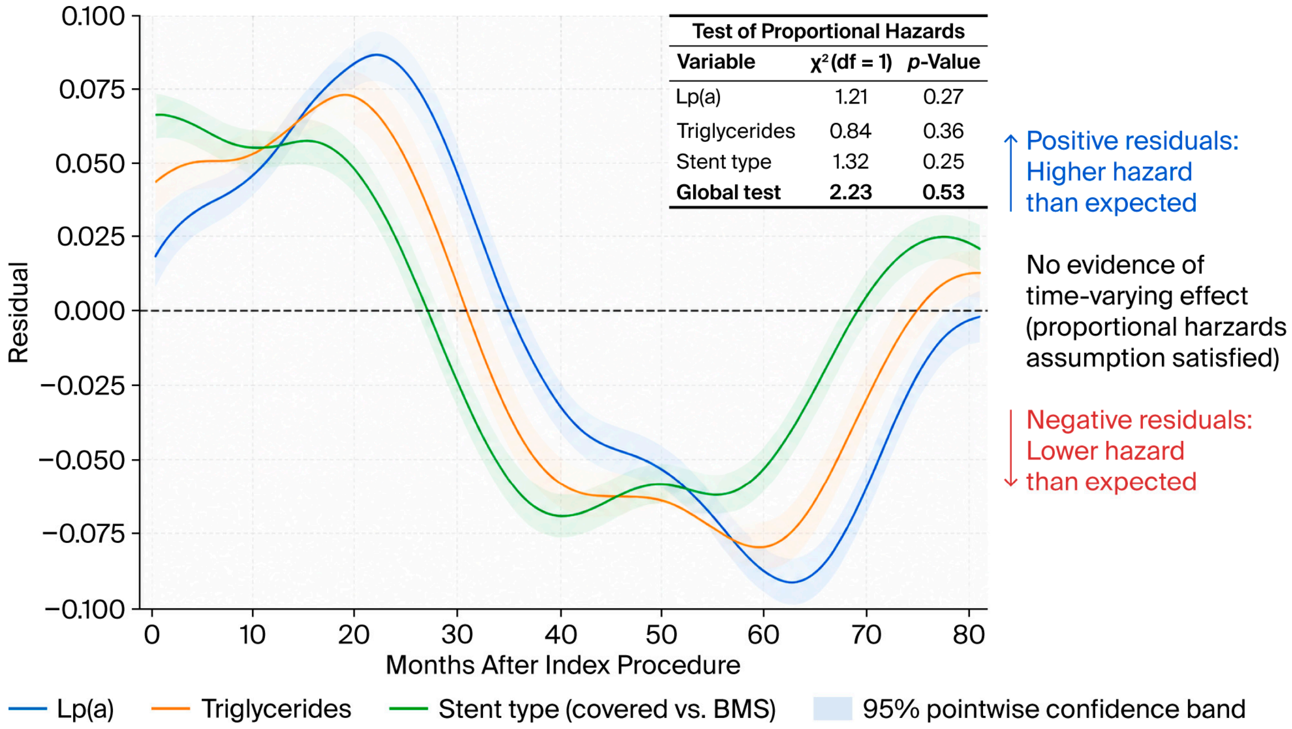

Figure S5. Scaled Schoenfeld residual assessment of the proportional hazards assumption. Smoothed scaled Schoenfeld residuals are plotted against follow-up time for the key covariates in the Cox proportional hazards model: Lp(a), triglycerides, and stent type. The horizontal dashed line at zero represents no time-varying covariate effect; residual curves fluctuating around zero without a systematic trend support the proportional hazards assumption. Shaded bands represent 95% pointwise confidence intervals around the smoothed residual curves. Positive residuals indicate a higher-than-expected hazard at that time point, whereas negative residuals indicate a lower-than-expected hazard. The inset table reports the Grambsch–Therneau proportional hazards test for each covariate and the global model test. No individual covariate showed evidence of a statistically significant time-varying effect: Lp(a), $\chi^2 = 1.21$, $p = 0.27$; triglycerides, $\chi^2 = 0.84$, $p = 0.36$; and stent type, $\chi^2 = 1.32$, $p = 0.25$. The global test was also non-significant, $\chi^2 = 2.23$, $p = 0.53$, supporting no detectable violation of the proportional hazards assumption. Abbreviations: BMS, bare-metal stent; CI, confidence interval; Lp(a), lipoprotein(a).
